# Biogenic Silver Nanoparticles Produced by Soil Rare Actinomycetes and Their Significant Effect on *Aspergillus*-derived mycotoxins

**DOI:** 10.3390/microorganisms11041006

**Published:** 2023-04-12

**Authors:** Mohamed N. Abd El-Ghany, Salwa A. Hamdi, Shereen M. Korany, Reham M. Elbaz, Ahmed N. Emam, Mohamed G. Farahat

**Affiliations:** 1 Botany and Microbiology Department, Faculty of Science, Cairo University, Giza 12613, Egypt; 2 Zoology Department, Faculty of Science, Cairo University, Giza 12613, Egypt; salwa_abdelhamid@cu.edu.eg; 3 Botany and Microbiology Department, Faculty of Science, Helwan University, Cairo 11795, Egypt; smkorany@pnu.edu.sa (S.M.K.); reham_elbaz@science.helwan.edu.eg (R.M.E.); 4 Department of Biology, College of Science, Princess Nourah bint Abdulrahman University, P.O. Box 84428, Riyadh 11671, Saudi Arabia; 5 Department of Biology, Faculty of Science, University of Bisha, P.O. Box 551, Bisha 61922, Saudi Arabia; 6 Refractories, Ceramics and Building Materials Department, Advanced Materials Technology & Mineral Resources Research Institute, National Research Centre (NRC), El Bohouth St., Dokki, Cairo 12622, Egypt; an.emam@nrc.sci.eg; 7 Nanomedicine & Tissue Engineering Research Lab, Medical Research Centre of Excellence, National Research Centre, El Bohouth St., Dokki, Cairo 12622, Egypt; 8 Biotechnology Department, Faculty of Nanotechnology for Postgraduate Studies, Cairo University, Sheikh Zayed Branch Campus, Giza 12588, Egypt

**Keywords:** biosynthesis, silver nanoparticles, mycotoxin production, antifungal activity, mycotoxin inhibition, rare actinomycetes, spore germination

## Abstract

The current investigation addressed the green synthesis of silver nanoparticles (AgNPs) using newly isolated silver-resistant rare actinomycetes, *Glutamicibacter nicotianae* SNPRA1 and *Leucobacter aridicollis* SNPRA2, and investigated their impact on the mycotoxigenic fungi *Aspergillus flavus* ATCC 11498 and *Aspergillus ochraceus* ATCC 60532. The formation of AgNPs was evidenced by the reaction’s color change to brownish and the appearance of the characteristic surface plasmon resonance. The transmission electron microscopy of biogenic AgNPs produced by *G. nicotianae* SNPRA1 and *L. aridicollis* SNPRA2 (designated Gn-AgNPs and La-AgNPs, respectively) revealed the generation of monodispersed spherical nanoparticles with average sizes of 8.48 ± 1.72 nm and 9.67 ± 2.64 nm, respectively. Furthermore, the XRD patterns reflected their crystallinity and the FTIR spectra demonstrated the presence of proteins as capping agents. Both bioinspired AgNPs exhibited a remarkable inhibitory effect on the conidial germination of the investigated mycotoxigenic fungi. The bioinspired AgNPs caused an increase in DNA and protein leakage, suggesting the disruption of membrane permeability and integrity. Interestingly, the biogenic AgNPs completely inhibited the production of total aflatoxins and ochratoxin A at concentrations less than 8 μg/mL. At the same time, cytotoxicity investigations revealed the low toxicity of the biogenic AgNPs against the human skin fibroblast (HSF) cell line. Both biogenic AgNPs exhibited feasible biocompatibility with HSF cells at concentrations up to 10 μg/mL and their IC_50_ values were 31.78 and 25.83 μg/mL for Gn-AgNPs and La-AgNPs, respectively. The present work sheds light on the antifungal prospect of the biogenic AgNPs produced by rare actinomycetes against mycotoxigenic fungi as promising candidates to combat mycotoxin formation in food chains at nontoxic doses.

## 1. Introduction

Nanotechnology is a rapidly growing, modern multidisciplinary science that involves the production and manipulation of matter at the nanoscale level. Compared with bulk materials, nanoparticles (less than 100 nm in at least one dimension) possess distinctive chemical and physical properties because of their vast surface area to volume proportion and high surface energy [1]. Owing to their admirable properties, metal nanoparticles have emerged as exploratory areas in physics, chemistry, materials science, drug delivery, the food sector, and biomedicine [2,3,4]. Among the metal nanoparticles, nanosilver has received considerable attention for its exceptional antibacterial, antifungal, anticancer, and antioxidant potential [5,6,7,8,9,10].

Generally, silver nanoparticles (AgNPs) are produced either by a “top-down” or “bottom-up” strategy. In the “top-down” approach, the bulk materials are broken down into small nanoparticles using various physical techniques, including ball milling and laser ablation [11,12,13], which are energy-consuming approaches that make them more capital-intensive. In the “bottom-up” strategy, AgNPs are synthesized via chemical reactions in which the self-assembly of atoms into nuclei occurs, further developing them into nanoscale particles. Fabrication of AgNPs by chemical methods involves the use of reducing agents, such as sodium borohydride, N,N-dimethyl formamide, and Tollens’ reagent [14]. Though being the most accustomed method for the synthesis of AgNPs, chemical methods are thought to cause environmental hazards due to using various perilous chemicals attributed to carcinogenicity, genotoxicity, and cytotoxicity [15]. Hence, the green synthesis of nanoparticles provides an alternative approach to conventional chemical and physical methods. The biosynthesis processes include the usage of bacteria, fungi, algae, or plant extracts, offering an eco-friendly green solution that reduces the utilization of toxic chemicals [16,17,18,19,20,21,22,23]. 

*Aspergillus* species are ubiquitous fungi that produce various life-threatening mycotoxins such as aflatoxins, ochratoxins, patulin, and citrinin in the contaminated food chains [24]. Aflatoxin is one of the carcinogenic and mutagenic mycotoxins predominantly produced by *A. flavus*, *A. parasiticus*, and *A. nomius*, whereas ochratoxin A (OTA) is a teratogenic and nephrogenic mycotoxin produced mainly by *A. ochraceus*, *A. westerdijkiae*, and *A. steynii* [25]. Of all the mycotoxins, aflatoxins are one of the most toxic and carcinogenic groups comprising four main types, namely, AFB1, AFB2, AFG1, and AFG2 [26]. On the other hand, OTA is responsible for various health issues due to its carcinogenicity, mutagenicity, hepatotoxicity, genotoxicity, immunotoxicity, embryotoxicity, and testicular toxicity [27].

Although the biosynthesis of AgNPs using various microorganisms is well-documented, there is limited research in the literature addressing the biosynthesis of AgNPs using actinomycetes (actinobacteria). The vast majority of described AgNP-producing actinomycetes belong to the genus *Streptomyces* [28,29,30,31,32,33,34,35], while the production of AgNPs by non-*Streptomyces* species (rare actinomycetes) is relatively unexplored. Herein, we address for the first time the biosynthesis of AgNPs by two rare actinomycetes, *G. nicotianae* SNPRA1 and *L. aridicollis* SNPRA2, and evaluate their antifungal and anti-mycotoxin efficacy on mycotoxigenic fungi at nontoxic doses.

## 2. Materials and Methods

### 2.1. Isolation of Actinobacteria

Soil samples were collected from the Wadi El-Natrun depression, Western desert, Egypt. The collected samples were subjected to dry heating at 100 °C for 15 min, which was followed by treatment with 1.5% phenol for 30 min at 30 °C. Subsequently, the physicochemically treated samples were serially diluted in sterile saline (NaCl, 9 g/L) and aliquots of each dilution (100 μL) were plated on humic-acid–vitamin agar (Kisan Biotech, Seoul, South Korea) plates supplemented with nalidixic acid (25 μg/mL) (Merck, Darmstadt, Germany) and cycloheximide (25 μg/mL) (Merck, Darmstadt, Germany) [36]. After incubation at 30 °C for 14 days, the developed colonies of different shapes were selected and purified by repeated subculturing. Afterwards, the purified colonies were subcultured on ISP2 agar (BD BBL and Difco, Franklin Lakes, NJ, USA) supplemented with various concentrations of AgNO_3_ (1–5 mM) and incubated at 30 °C for 14 days. Accordingly, the resistant isolates were picked and assessed for the production of biogenic AgNPs.

### 2.2. Identification and Phylogenetic Analysis

The promising AgNP-producing strains SNPRA1 and SNPRA2 were identified based on their 16S rRNA gene sequences. Briefly, the 16S rRNA gene was amplified by polymerase chain reaction (PCR) using the universal primers 27F and 1492R as described elsewhere [37]. The nucleotide sequences of the purified amplicons were sequenced using an ABI 3730XL sequence analyzer (Applied Biosystems, Waltham, MA, USA). A sequence similarity search was performed using The NCBI BLASTN and the EzTaxon-e server database [38]. A phylogenetic tree was constructed in the context of the 16S rRNA gene sequences using the neighbor-joining method of the MEGA-X software (version 10.0.5), and the bootstrap analysis was performed based on 1,000 replicates.

### 2.3. Biosynthesis of AgNPs

The biosynthesis of AgNPs was conducted by using AgNO_3_ (Alfa Aesar, Ward Hill, MA, USA) as a metal precursor and the actinomycete biomass extract as the reducing and stabilizing agent. Typically, each actinobacterial isolate was inoculated in a conical flask containing 50 mL of ISP2 broth (g/L: glucose 4.0, yeast extract 4.0, and malt extract 10.0) and incubated at 30 °C and 150 rpm for 96 h. Subsequently, the culture was centrifuged at 12,000× *g* for 15 min, and the collected cells were washed twice with sterile distilled water. Then, the washed biomass was resuspended in 50 mL of ultrapure water (18.2 Mcm) and incubated on a shaking incubator (150 rpm) at 30 °C for 72 h. After osmotic lysis, the cell lysates were filtered through Whatman No. 1 filter papers yielding actinomycete biomass extracts. After that, the extract was mixed with an equal volume of 2 mM AgNO_3_ solution, and the mixture was incubated at 30 °C and 150 rpm for 48 h under dark conditions. The synthesis was monitored by visual inspection for the color change into brown as the initial indicator of AgNP biosynthesis, which was confirmed by UV–Vis absorbance of the reaction mixture. For purification of the biogenic AgNPs, the brownish mixture was centrifuged at 40,000× *g* for 15 min using a Sorvall LYNX 6000 centrifuge (Thermo Scientific, Waltham, MA, USA). The AgNP pellets were resuspended in ultrapure water and centrifuged again at 40,000× *g* for 15 min. To remove any undesirable impurities, the pellets were washed three more times. Finally, the nanosilver pellets were dried in a vacuum oven at 50 °C for 24 h. Based on the potent reduction of AgNO_3_ into AgNPs, two proficient isolates designated SNPRA1 and SNPRA2 were selected and used for further characterization.

### 2.4. Characterization of Biogenic AgNPs

UV–Vis absorbance spectra of the biogenic AgNPs were recorded in the 300–700 nm range with a resolution of 1 nm using an Epoch 2 UV–Vis spectrophotometer (BioTek, Santa Clara, CA, USA). The size and shape of biogenic AgNPs were investigated by high-resolution transmission electron microscopy (HRTEM) using a JEM-2100 transmission electron microscope (JEOL, Tokyo, Japan) with a 200 kV acceleration voltage. The X-ray diffraction (XRD) patterns of biogenic AgNPs were analyzed on a D8 Discover X-ray diffractometer (Bruker, Karlsruhe, Germany). The Cu Kα radiation with a current of 30 mA and applied voltage of 40 kV was used as a light source. The 2θ values were measured with a scan speed of 0.5°/min in a range from 20 to 90°. Fourier-transform infrared (FTIR) spectroscopy was performed using a Nicolet 6700 FT-IR spectrometer (Thermo Scientific, Waltham, MA, USA), and the scanning spectra were recorded within the range of 4000 to 400 cm^−1^. 

### 2.5. Antifungal Activity

The inhibitory effect of the biogenic AgNPs on the conidial germination of the mycotoxigenic fungi *A. flavus* (ATCC 11498) and *A. ochraceus* (ATCC 60532) was investigated in vitro. In brief, conidia of *A. flavus* and *A*. *ochraceus,* cultivated on potato dextrose agar (PDA) plates (Condalab, Madrid, Spain) at 28 °C for 7 days, were harvested, and suspended in sterile saline solution (NaCl, 0.9 %). After filtration through sterile muslin, the conidial count was adjusted to 2 × 10^6^ conidia/mL using a hemocytometer. Subsequently, 50 µL of the prepared conidial suspension was transferred to each well of a 96-well microtiter plate containing 100 μL of potato dextrose broth (PDB) (Condalab, Madrid, Spain) amended with different concentrations of AgNPs. Wells containing AgNP-free PDB served as the negative control. Then, the microtiter plates were incubated at 28 °C for 16 h. Afterward, the percentage of germinated conidia was calculated by analyzing 100 conidia under a DM500 optical microscope (Leica, Heerbrugg, Switzerland). A conidium was considered germinated if the germ tube length was equal to or longer than the conidial length. The minimum inhibitory concentration (MIC) values of AgNPs were estimated statistically using the GraphPad Prism software (version 7.0.0). The MIC values indicating the lowest concentration of biogenic AgNPs inhibiting conidial germination were expressed as µg/mL. Moreover, leakage of proteins and DNA in AgNPs-treated *A. flavus* and *A*. *ochraceus* was investigated following the method described elsewhere [39].

### 2.6. Effect of Biogenic AgNPs on Mycotoxin Production

The impact of biogenic AgNPs on the production of total aflatoxins and OTA by *A. flavus* and *A. ochraceus*, respectively, was assessed. Practically, the conidia of each investigated mycotoxigenic fungi were inoculated into yeast extract sucrose (YES) broth ((20% sucrose and 2% yeast extract (Merck, Darmstadt, Germany)) containing various concentrations of actinomycete-mediated biogenic AgNPs [40]. Experiments containing sterile distilled water instead of the biogenic AgNPs were considered to be the negative control. After incubation for 10 days at 28 °C, the produced total aflatoxins (AFLAs) and OTA were determined using Celer^®^ AFLA and Celer^®^ OCHRA quantitative ELISA test kits (Eurofins technologies, Budapest, Hungary), respectively.

### 2.7. Cytotoxicity Assay

The cytotoxicity of the as-prepared biogenic AgNPs was investigated in vitro using human skin fibroblast (HSF) cells. The HSF cell line was kindly provided by Nawah Scientific Inc. (Mokatam, Cairo, Egypt). The HSF cells were cultured and maintained in the Dulbecco’s modified eagle medium (DMEM) containing 10% heat-inactivated fetal bovine serum (Gibco; Thermo Fisher Scientific, Loughborough, UK), streptomycin (100 µg/mL), and penicillin (100 units/mL). The cell line was cultured under 5% CO_2_ in a water-jacketed incubator (BINDER GmbH, Tuttlingen, Germany) in a high-humidity atmosphere at 37 °C. The potential cytotoxicity of the bio-fabricated AgNPs was assayed against HSF cells using the sulforhodamine B (SRB) method [41]. 

### 2.8. Statistical Analysis

The presented data are the means of triplicate assays. The analysis of variance (ANOVA) was conducted, and the significant differences were compared via Duncan’s test using the IBM SPSS software (version 22), with the critical difference set at a 5% level of probability. The statistical linear regression and the half-maximal inhibitory concentration (IC_50_) were executed using GraphPad Prism software (version 7.0.0).

## 3. Results

### 3.1. Isolation of Actinobacteria

Fifty-eight actinobacterial isolates were recovered from arid soil samples obtained from the Egyptian desert and screened for their capability to tolerate AgNO_3_. The preliminary screening revealed that the assessed actinomycete isolates tolerated the presence of Ag^+^ up to 3 mM, but none tolerated 4 mM of AgNO_3_. Of the screened fifty-eight isolates, nineteen were tolerant to 1 mM of AgNO_3_, eleven isolates were tolerant to 2 mM, and five isolates were tolerant to 3 mM. Consequently, the most tolerant five isolates were selected for further investigations.

### 3.2. Identification and Phylogenetic Analysis

**Figure 1:**
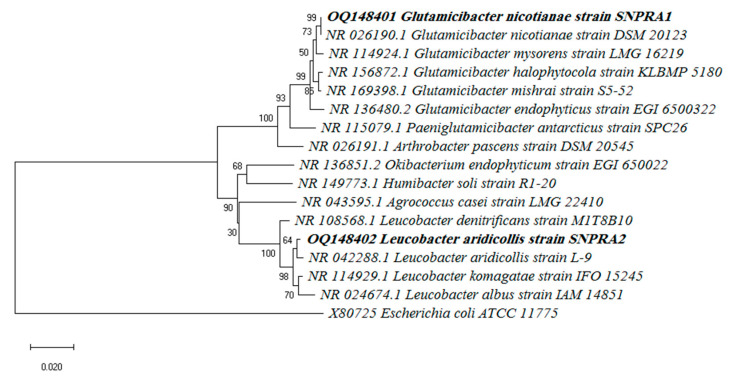
Phylogenetic tree showing the relationships between *G. nicotianae* SNPRA1 and *L. aridicollis* SNPRA2 and the most closely related species.

The selected strains were identified based on their 16S rRNA gene sequences. The BLAST results of the 16S rRNA gene revealed that the strain SNPRA1 shared a 99.78% identity with the *G. nicotianae* strain DSM 20123 (accession number: NR_026190.1), a 99.34% identity with the *G. mysorens* strain LMG 16219 (accession number: NR_114924.1), and a 99.26% identity with the *G. halophytocola* strain KLBMP 5180 (accession number: NR_156872.1). On the other hand, the strain SNPRA2 shared a 99.43% identity with the *L. aridicollis* strain L-9 (accession number: NR_042288.1), a 99.07% identity with the *L. komagatae* strain IFO 15245 (accession number: NR_114929.1), and a 98.63% with the *L. denitrificans* strain M1T8B10 (accession number: NR_108568.1). Based on their 16S rRNA gene sequences, the strains SNPRA1 and SNPRA2 were putatively identified as *G. nicotianae* and *L. aridicollis*, respectively. The 16S rRNA gene sequences of the strains SNPRA1 and SNPRA2 were submitted into the GenBank and accession numbers OQ148401 and OQ148402 were assigned, respectively. The neighbor-joining phylogenetic tree was constructed in the context of the 16S rRNA gene sequences (Figure 1).

### 3.3. Biosynthesis of AgNPs

The biomass extracts of the selected isolates were evaluated for their ability to synthesize AgNPs. Of the five Ag-tolerant isolates, only two strains exhibited the color of the AgNO_3_ solution changing into brown, and this formation of AgNPs was confirmed by spectrophotometric scanning of the reaction mixture. As a result, the two strains, designated SNPRA1 and SNPRA2, demonstrating the characteristic surface plasmon resonance (SPR) at 400-430 nm (indicating the potential for the production of AgNPs) were selected for further investigations. The control experiments without biomass extracts did not produce any change in color after the same incubation period.

### 3.4. Characterization of Biogenic AgNPs

**Figure 2:**
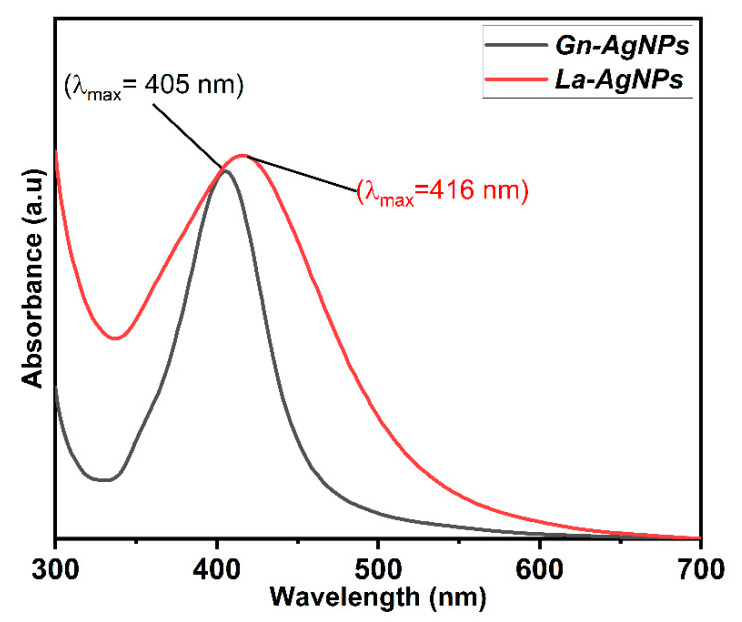
UV–Vis spectra of Gn-AgNPs and La-AgNPs produced by *G. nicotianae* SNPRA1 and *L. aridicollis* SNPRA2, respectively.

**Figure 3:**
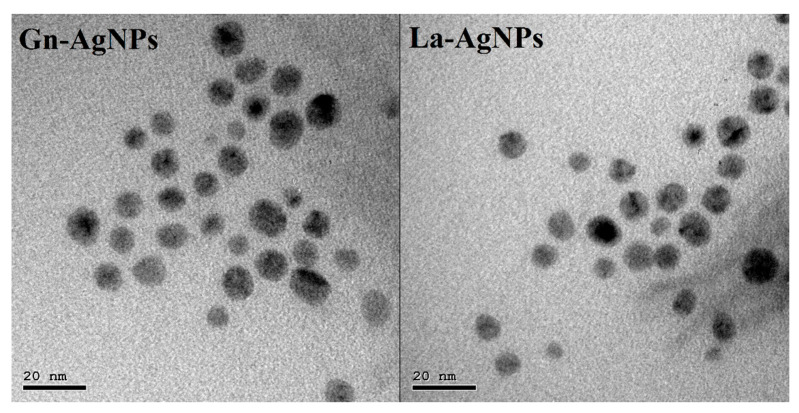
Transmission electron micrographs showing the size and shape of Gn-AgNPs and La-AgNPs produced by *G. nicotianae* SNPRA1 and *L. aridicollis* SNPRA2, respectively. The average sizes of Gn-AgNPs and La-AgNPs were 8.48 ± 1.72 nm and 9.67 ± 2.64 nm, respectively.

**Figure 4:**
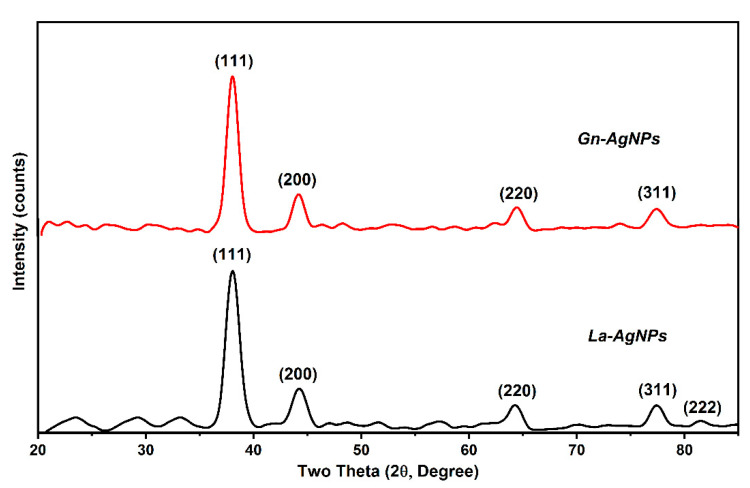
XRD spectra of the biogenic Gn-AgNPs and La-AgNPs produced by *G. nicotianae* SNPRA1 and *L. aridicollis* SNPRA2, respectively.

**Figure 5:**
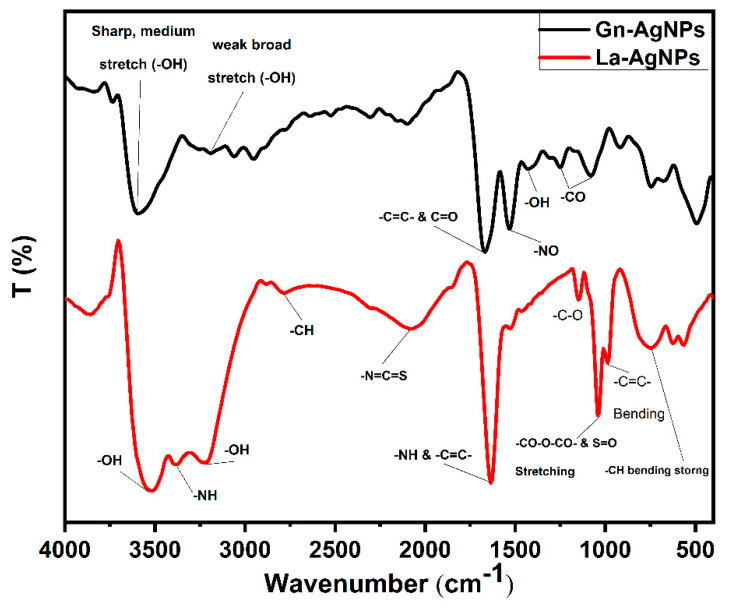
FTIR spectra of the biogenic Gn-AgNPs and La-AgNPs produced by *G. nicotianae* SNPRA1 and *L. aridicollis* SNPRA2, respectively.

The biosynthesis of AgNPs was determined by the reaction’s color change to brownish, which is the primary indication of AgNP formation. The biogenic AgNPs produced by the biomass extracts of *G. nicotianae* SNPRA1 and *L. aridicollis* SNPRA2 were designated as Gn-AgNPs and La-AgNPs, respectively. UV–Vis spectra of the biogenic Gn-AgNPs and La-AgNPs revealed characteristic absorption peaks located at 405 and 416 nm (Figure 2), respectively, which were in agreement with the surface plasmon resonance reported for AgNPs. In addition, the morphological properties based on TEM analysis confirmed the generation of monodispersed spherical AgNPs by both actinomycete biomass extracts. The average sizes of the Gn-AgNPs and La-AgNPs, as determined by TEM, were 8.48 ± 1.72 nm and 9.67 ± 2.64 nm, respectively (Figure 3). Furthermore, the crystallographic structures of the biogenic Gn-AgNPs and La-AgNPs were confirmed by XRD analysis. The XRD patterns revealed four intense peaks in the whole spectra, with 2θ peak values around 38.26°, 44.47°, 64.71°, and 77.73° (Figure 4). The spectra correlated to the main characteristic peaks of AgNPs (COD card No./file No. 1509146) corresponding to the lattice plane clusters of (111), (200), (220), and (311). The obtained lattice plane values confirmed the face-centered cubic (FCC) nature of the biogenic AgNPs. Finally, the surface properties of the as-prepared spherical-like shaped La-AgNPs and Gn-AgNPs were investigated via diffused reflectance as a function of wavenumber by FTIR. The obtained spectra showed a wide variety of surface functional groups that decorated the silver nanoparticles’ surfaces, which was based on the use of actinomycetes in the formation of the biogenic silver nanoparticles (Figure 5).

### 3.5. Antifungal Activity

**Figure 6:**
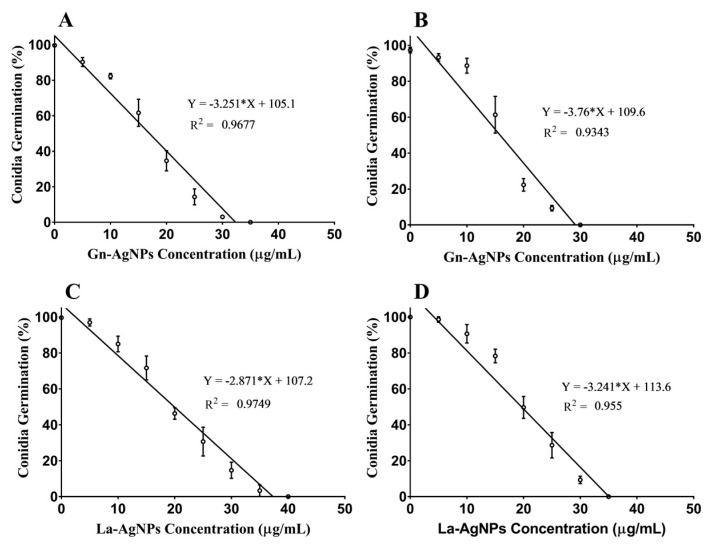
Effect of the biogenic AgNPs on conidial germination. (**A**) *A. flavus* treated with Gn-AgNPs, (**B**) *A*. *ochraceus* treated with Gn-AgNPs, (**C**) *A. flavus* treated with La-AgNPs, (**D**) *A*. *ochraceus* treated with La-AgNPs. Error bars represent the standard deviation of triplicate assays.

**Figure 7:**
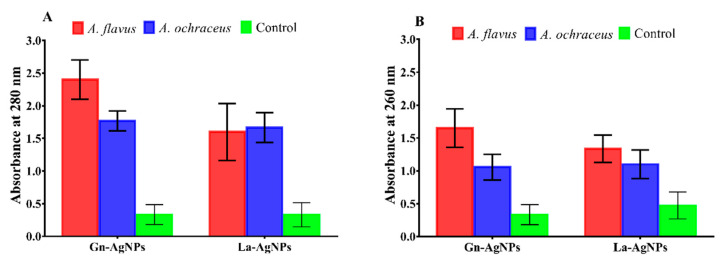
Effect of the biogenic AgNPs on the leakage of proteins (**A**) and DNA (**B**) from the conidia of *A. flavus* and *A*. *ochraceus* compared with the untreated control.

The antifungal potential of the actinomycete-mediated biogenic AgNPs was evaluated against two mycotoxin-producing fungi. Our findings provide evidence of the inhibitory effect of Gn-AgNPs and La-AgNPs in the conidial germination of both mycotoxigenic fungi (Figure S1). The MIC values of Gn-AgNPs and La-AgNPs against *A. flavus* and *A*. *ochraceus* were statistically deduced (Figure 6). The results revealed a slightly superior inhibitory effect of Gn-AgNPs compared with that of La-AgNPs. The deduced MIC values of Gn-AgNPs and La-AgNPs against *A. flavus* were 32.34 and 37.35 μg/mL, respectively. In the case of *A*. *ochraceus*, the deduced MIC values of Gn-AgNPs and La-AgNPs were 29.15 and 35.06 μg/mL, respectively. Furthermore, the present results indicate that the exposure of *A. flavus* and *A*. *ochraceus* conidia to the as-prepared biogenic AgNPs led to the significant leakage of DNA and proteins (Figure 7).

### 3.6. Effect of Biogenic AgNPs on Mycotoxin Production

**Figure 8:**
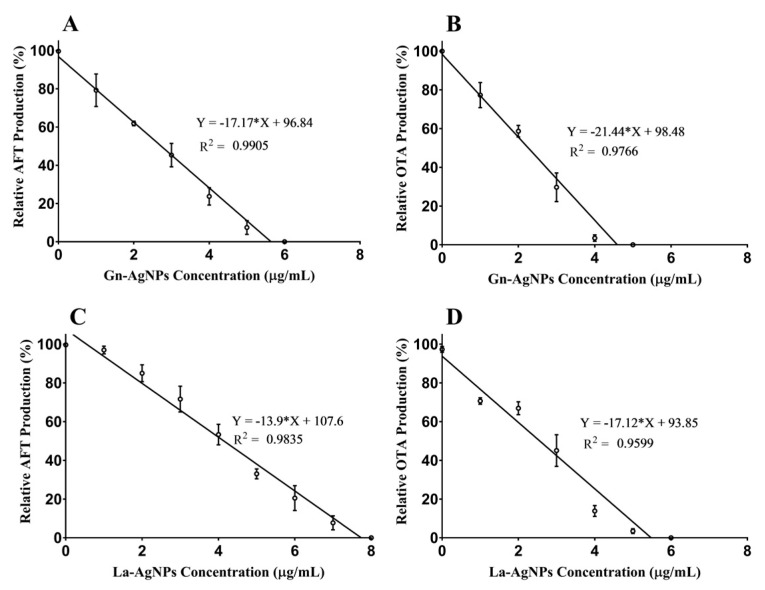
Linear regression model fit showing the anti-mycotoxin activity of the biogenic AgNPs. (**A**) *A. flavus* treated with Gn-AgNPs, (**B**) *A*. *ochraceus* treated with Gn-AgNPs, (**C**) *A. flavus* treated with La-AgNPs, (**D**) *A*. *ochraceus* treated with La-AgNPs. Error bars represent the standard deviation of triplicate assays.

The anti-mycotoxin activity of actinobacteria-mediated biogenic AgNPs was evaluated against aflatoxigenic *A. flavus* ATCC 11498 and ochratoxigenic *A. ochraceus* ATCC 60532. The anti-mycotoxin activity of AgNPs was determined by comparing the relative toxin production of the treated fungi with that of the untreated control. The results revealed an obvious declining trend in the total AFLA and OTA contents in the treated mycotoxigenic cultures (Figure 8). In this study, Gn-AgNPs significantly decreased the total AFLA and OTA content in treated *A. flavus* ATCC 11498 and *A. ochraceus* ATCC 60532, respectively, in a dose-dependent manner. Apparently, Gn-AgNPs completely inhibited the total AFLA and OTA production at concentrations of 5.64 and 4.59 μg/mL, respectively. Likewise, La-AgNPs completely inhibited the total AFLA and OTA production by *A. flavus* ATCC 11498 and *A. ochraceus* ATCC 60532 at concentrations of 7.74 and 5.48 μg/mL, respectively.

### 3.7. Cytotoxicity Assay

**Figure 9:**
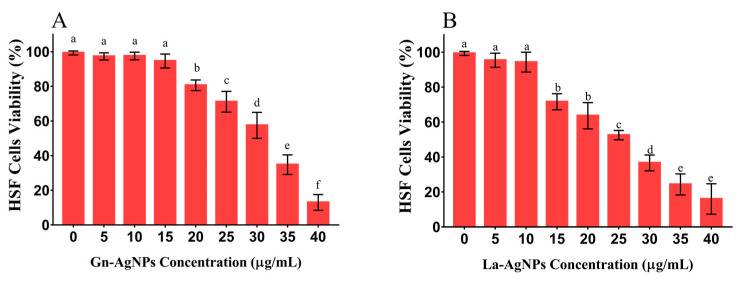
SRB cell viability assay for the determination of the cytotoxicity of the biogenic Gn-AgNPs (**A**) and La-AgNPs (**B**) against HSF cell lines. Columns headed by the same letter were not significantly different according to Duncan’s multiple range test (*p* < 0.05). Error bars represent standard deviations.

In the present investigation, the potential cytotoxicity of the actinomycete-mediated biogenic AgNPs was tested in vitro against HSF cell lines. The results obtained from the SRB assay clarified the biocompatibility of both investigated AgNPs with HSF cells at concentrations up to 10 μg/mL (Figure 9). Regarding their cytotoxicity, Gn-AgNPs did not exhibit any significant differences in the viability of the treated HSF cells when exposed to concentrations up to 15 μg/mL, as compared with the untreated control. A further increase in the Gn-AgNP concentration significantly reduced the cell viability. Indeed, the La-AgNPs demonstrated a slightly higher cytotoxicity effect on HSF cells. However, no significant difference in the HSF cell viability was observed up to 10 μg/mL, as compared with the untreated control. The results revealed that the IC_50_ values of Gn-AgNPs and La-AgNPs against HSF cells were 31.78 and 25.83 μg/mL, respectively.

## 4. Discussion

Recently, the green synthesis of nanoparticles has attracted more attention as an emerging eco-friendly strategy due to its high efficiency, nontoxicity, and environmental protection [41]. Consequently, our study sheds light on the pronounced antifungal and anti-mycotoxin potential of biogenic AgNPs produced by two rare actinomycetes. In this investigation, we used an integrated method to isolate non-*Streptomyces* actinomycetes via the pretreatment of soil samples at 100 °C for 15 min, which was followed by treatment with 1.5% phenol for 30 min at 30 °C. These physical and chemical pretreatments could eliminate most fungi, streptomycetes, and other common bacteria. It is believed that pretreatment of environmental samples by drying and heating stimulates the isolation of rare actinomycetes. In a previous study, the pretreatment of samples at 100 °C for 15 min enabled the isolation of rare actinomycetes belonging to the genera *Pseudonocardia*, *Blastococcus*, *Nocardiopsis*, *Actinocorallia*, *Micromonospora*, *Dactylosporangium*, and *Streptosporangium* [42]. Subramani and Aalbersberg reviewed how the dry heating of samples, followed by treatment with certain chemicals such as 1.5% phenol, 0.01% benzethonium chloride, 0.05% sodium dodecylsulfate (SDS), and 0.03% chlorhexidine gluconate, drastically eliminated most microorganisms and unwanted actinomycetes propagules belonging to the *Streptomyces* species [43]. Moreover, it has been suggested that the pretreatment of the soil samples with 1.5% phenol (30 °C for 30 min) inhibits the growth of fungi, bacteria, and other common actinomycetes by denaturing their proteins or by disrupting their cell membrane, and facilitates the recovery of phenol-resistant actinomycetes [44]. Likewise, various rare actinomycetes belonging to the genera *Gordonia*, *Nonomuraea*, *Actinoplanes*, *Microbispora*, and *Micromonospora* were selectively isolated from soil samples and subjected to harsher pretreatments, including moist (50 °C for 6 min) and dry (120 °C for 1 h) heating and 1.5 % phenol [45]. Herein, the biomass extracts of Ag-resistant rare actinobacteria *G. nicotianae* SNPRA1 and *L. aridicollis* SNPRA2 reduced AgNO_3_ and yielded biogenic AgNPs under dark conditions. To the best of our knowledge, there is limited data on the biogenic AgNPs from desert-derived non-*Streptomyces* actinomycetes, and to date, no published reports describe the fabrication of AgNPs using microorganisms belonging to the *Glutamicibacter* and *Leucobacter* genera. Nonetheless, previous reports indicated the metal resistance in *Glutamicibacter* and *Leucobacter* spp. It has been suggested that heavy metals exert selective pressure on the exposed microbial communities and lead to the evolution of metal resistance determinants to sequester and transform these compounds [46,47,48]. In this context, it has been reported that metal-resistant *G. nicotianae* MSSRFPD35 exhibited the potential to grow and degrade phenol in the presence of several heavy metals such as Pb, Ni, Cd, Co, and Cu [49]. Likewise, various species of the genus *Leucobacter*, which belongs to the phylum *Actinobacteria*, can tolerate a wide variety of heavy metals such as Cd, Cr, Cu, As, and Pb [50,51,52]. In agreement with our findings, the biomass extract from the Ag-tolerant actinobacterium *Nocardiopsis dasonvillei* KY772427 was used as a reducing agent for the biosynthesis of bioactive AgNPs [53]. It has commonly been assumed that the biosynthesis of metal nanoparticles is correlated to the capability of microorganisms for tolerating heavy metals [54]. The exact mechanism of the biosynthesis of metal nanoparticles by microorganisms is not fully understood; however, it has been suggested that microbial extracts could contain biomolecules such as polyphenols, polysaccharides, proteins, vitamins, and enzymes that have the potential to reduce silver salts and convert them into AgNPs [55]. Moreover, a recent study proved the capability of nicotinamide adenine dinucleotide phosphate (NADPH) to reduce silver nitrate as the sole reducing agent forming AgNPs [56]. 

In this work, the emergence of the brown color denoted the reduction in AgNO_3_ and the fabrication of AgNPs [32,35,57,58]. The biosynthesis of Gn-AgNPs and La-AgNPs was affirmed by the appearance of single absorption peaks at 405 and 416 nm, respectively, which could be attributed to the characteristic SPR of AgNPs. Generally, distinctive AgNPs demonstrate characteristic SPR at wavelengths ranging from 400 to 450 nm [33,59,60,61,62,63]. The current study demonstrated the formation of pure Gn-AgNPs and La-AgNPs with a crystalline nature through XRD patterns that illustrated the distinguished characteristic diffraction peaks, which were consistent with previous reports on AgNPs [30,64,65,66,67,68]. In addition, the FTIR spectra revealed the presence of biomolecules, especially proteins, on the surface of Gn-AgNPs and La-AgNPs. In addition to their reducing properties, the biomass extracts of *G. nicotianae* SNPRA1 and *L. aridicollis* SNPRA2 seem to play a crucial role as stabilizing and capping agents that may confer a bifold action regarding the fabrication of biogenic AgNPs. This finding was in line with the previous literature and concords with the presence of protein capping on the surface of biogenic AgNPs that stabilizes the nanoparticles in aqueous environments [69,70,71,72,73]. It has been assumed that proteins can bind to nanoparticles as capping agents through their cysteine residues and/or free amine groups [74]. It is worth mentioning that the biomass extracts of *G. nicotianae* SNPRA1 and *L. aridicollis* SNPRA2 reduced Ag^+^ to Ag^0^ under dark conditions without photocatalysis. On the contrary, the biosynthesis of AgNPs by the actinomycete *Sinomonas mesophila* MPKL 26 was achieved only after exposure to sunlight [75]. Additionally, the sunlight-assisted biosynthesis of AgNPs by various fungal and plant extracts is well-documented [76,77,78,79,80,81].

In this study, the biogenic Gn-AgNPs and La-AgNPs exhibited a remarkable inhibitory effect on the conidial germination of the investigated mycotoxigenic fungi, which was accomplished with impaired membrane integrity evidenced by the leakage of DNA and cellular proteins. In the same way, Khalil and coworkers proved that biogenic AgNPs produced by *Penicillium chrysogenum* NG85 and *Fusarium chlamydosporum* NG30 exert their antifungal activity against *A. flavus* and *A. ochraceous* via cellular membrane damage [39]. Aspergilli and many fungi produce conidia “asexual spores” for dispersion or to survive hostile conditions [82,83]. As conidial germination is the first step for the development of *Aspergillus* spp., attempts to prevent their germination could diminish the adverse impact of these widespread fungi. These findings are in harmony with previous studies suggesting that AgNPs provoke the permeability of membranes, causing the leakage of DNA and proteins [84,85,86,87]. Similarly, biogenic AgNPs produced by the *Pseudomonas poae* strain CO showed a significant inhibitory effect on the spore germination of *F. graminearum* [88]. Hence, the inhibition of spore/conidial germination by AgNPs could be attributed to reducing the fungal propagation, and thus, their risk. Additionally, biogenic AgNPs produced by *F. oxysporum* inhibited the sclerotial germination of *Stromatinia cepivora* [89,90]. It has been suggested that the antifungal activity of nanosilver may be attributed to the attachment of AgNPs to the cell wall and the anchoring of the cell membranes causing damage and leakage to the intracellular content, which eventually leads to cell death [55,91]. Furthermore, AgNPs were reported to cause surface protein damage, nucleic acid damage, and the blockage of proton pumps [92]. In addition, several possible modes of action have been elucidated regarding the antifungal activity of AgNPs. The extracellular accumulation of AgNPs is thought to cause a dynamic release of Ag^+^ that penetrates the cell, leading to an accumulation of intracellular reactive oxygen species (ROS) which hurt the proteins of the membrane and affect the reactions of electron transport, triggering apoptosis [93]. Furthermore, Ag^+^ and AgNPs modulate the transcriptome and metabolome, altering the essential functions of fungal cells. It has been reported that Ag^+^ and AgNPs cause the downregulation of the tricarboxylic acid cycle genes and other genes involved in ergosterol synthesis and lipid metabolism, leading to structural alternations principally at the level of biological membranes [94]. 

Mycotoxigenic fungi such as *A. flavus* and *A. ochraceus* have a detrimental effect on economic plants such as rice plants because they cause biotic stress and significantly decrease the plant’s physiological activity [95]. Additionally, aflatoxins and OTA are carcinogenic secondary metabolites that are mainly produced by *A. flavus* and *A. ochraceus*, respectively [96]. These mycotoxins may be considered more dangerous than the fungi themselves, making it necessary to study the effect of AgNPs on the mycotoxin production by *A. flavus* and *A. ochraceus* [97]. Thus, the determination of the MIC of the AgNPs that inhibit mycotoxin production completely is highly recommended, and its value should be lower than that required for fungal growth inhibition [98,99]. Our results revealed that the MIC values required for the complete inhibition of total aflatoxin production by *A. flavus* or OTA production by *A. ochraceus* were much lower than that required for the total inhibition of fungal spore germination. In this regard, it has been reported that the biogenic AgNPs produced by *A. terreus* and *P. expansum* exhibited a 58.87 and 52.18% reduction of OTA production, respectively [100]. In good agreement with these findings, the deduced MIC values for the complete inhibition of total aflatoxin production by *A. flavus* using biosynthesized AgNPs from *F. chlamydosporum* NG30 and *P. chrysogenum* NG85 were 5.8 or 5.5 µg/mL, respectively. Meanwhile, the MIC values found for the previously synthesized AgNPs from *F. chlamydosporum* NG30 and *P. chrysogenum* NG85 that inhibited the production of OTA by *A. ochraceus* were 6.3 and 6.1 µg/mL, respectively [39]. Although having superior antimicrobial activity, nanosilver is causing concerns regarding its negative impact on human health due to its potential toxicity [101,102,103]. Thus, we evaluated the cytotoxicity of the as-prepared biogenic AgNPs against HSF cell lines. Based on the obtained results, no significant cytotoxic effect was observed in HSF cells upon exposure to Gn-AgNPs and La-AgNPs in concentrations up to 10 and 15 µg/mL, with IC_50_ values of 31.78 and 25.83 μg/mL, respectively. At the same time, Gn-AgNPs and La-AgNPs completely inhibited mycotoxin production at concentrations lower than 8 µg/mL. By comparing the effective anti-mycotoxin concentrations with cytotoxic ones, we suggest that Gn-AgNPs and La-AgNPs could be used as safe anti-mycotoxin agents at nontoxic doses. 

## 5. Conclusions

The silver-resistant rare actinomycetes, *G. nicotianae* SNPRA1 and *L. aridicollis* SNPRA2, could be promising candidates for the biosynthesis of AgNPs in a facilitated eco-friendly process. The actinomycete-mediated AgNPs could disrupt the life cycle of the mycotoxigenic fungi by inhibiting their conidial germination, which is the first step in fungal propagation. The data presented here provide evidence that biogenic AgNPs induce the leakage of cellular proteins and DNA, reflecting the disruption of membrane integrity. Furthermore, the bioinspired AgNPs completely inhibited the production of total aflatoxins and ochratoxin A in *A. flavus* and *A. ochraceus* at nontoxic doses. Owing to their biocompatibility and low toxicity, the as-prepared biogenic AgNPs produced by desert-derived actinomycetes could be used as potent antifungals and anti-mycotoxins at nontoxic doses.

## Data Availability

The essential data supporting the reported results are contained in this study. All other data are available upon request from the corresponding authors.

## Supplementary Materials

The following supporting information can be downloaded at: https://www.mdpi.com/article/10.3390/microorganisms11041006/s1, Figure S1: Effect of the biogenic AgNPs on the conidial germination. The percentage of germinated conidia was calculated by analyzing 100 conidia under an optical microscope. A conidium was considered germinated if the germ tube length was equal to or longer than the conidial length.
