# Supplementary material for: Biogenic Silver Nanoparticles Produced by Soil Rare Actinomycetes and Their Significant Effect on Aspergillus-derived mycotoxins
Source: Microorganisms. 2023 Apr 12;11(4):1006. doi: 10.3390/microorganisms11041006 (PMC10142716; doi:10.3390/microorganisms11041006)
Supplement: Supplementary file 1 [file microorganisms-11-01006-s001.zip › microorganisms-2291807-supplementary.pdf]

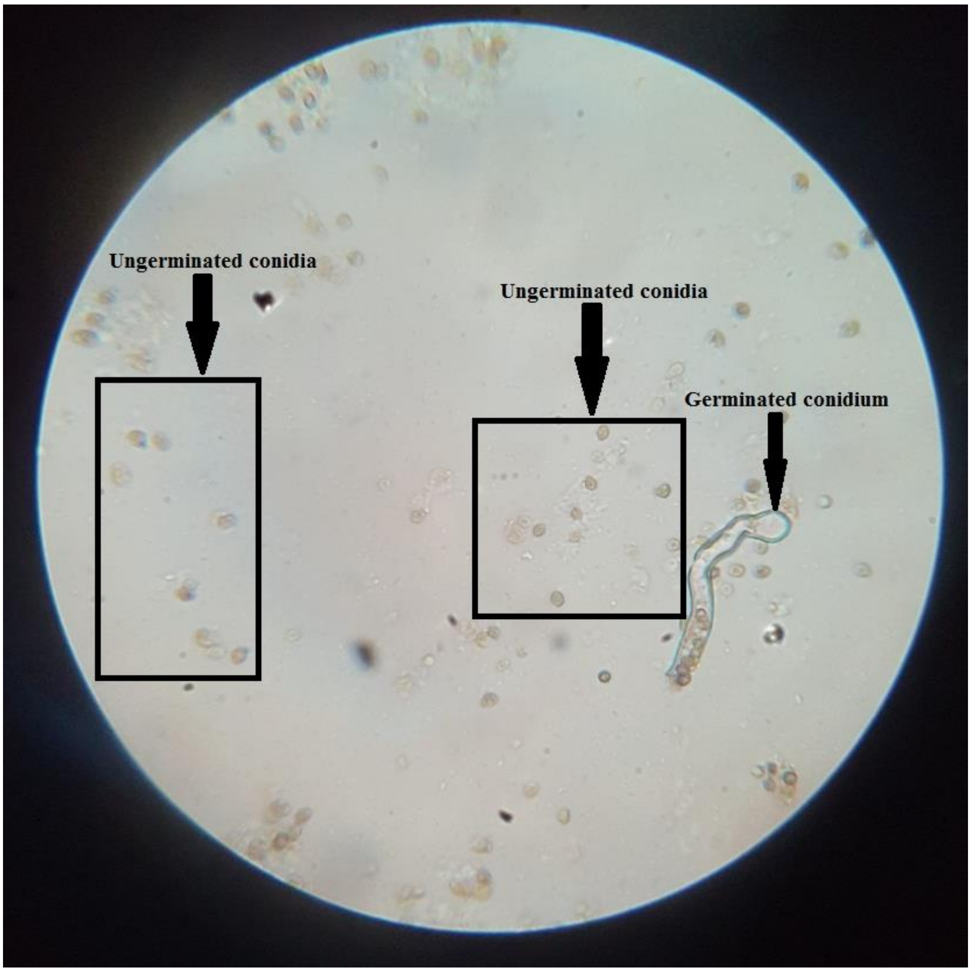

Figure S1: Effect of the biogenic AgNPs on the conidial germination (representative figure). The percentage of germinated conidia was calculated by analyzing 100 conidia under an optical microscope. A conidium was considered germinated if the germ tube length was equal to or longer than the conidial length.
